# A real-time ripeness detection model for tomatoes in complex greenhouse environments

**DOI:** 10.3389/fpls.2026.1853690

**Published:** 2026-05-28

**Authors:** Bo Cheng, Xuli Wu, Hao Du, Xiangying Qi, Junhui Wu, Shumei Zhao

**Affiliations:** 1 College of Water Resources and Civil Engineering, China Agricultural University, Beijing, China; 2 College of Agriculture, Shihezi University, Shihezi, China; 3 College of Landscape Architecture and Tourism Management, Xinjiang Vocational and Technical College of Applied Technology, Kuitun, China

**Keywords:** attention mechanism, greenhouse tomato, real-time detection, ripeness detection, YOLOv11

## Abstract

Timely harvesting of fresh tomatoes is urgently needed. To address this issue, this study proposes DDC-YOLOv11n, a model suitable for real-time detection of tomato ripeness in complex greenhouse environments. A Zero-DCE adaptive enhancement module is first deployed at the input stage to restore and enhance the true color and texture details of the images. An improved Deep Residual Shrinkage Network (DRSN) is then added to YOLOv11n to perform adaptive soft-threshold filtering on feature maps, reducing the interference of image noise on the detection targets. Finally, the CBAM spatial attention is enhanced through dilated convolution and channel grouping to form the LKCBAM module, which expands the equivalent receptive field while controlling the increase in parameters, thereby improving tomato detection accuracy in occluded and dense scenes. Experimental results show that the DDC-YOLOv11n model achieves the best recognition performance: compared with the original YOLOv11n, its mAP@0.5, precision, recall, and F1 score are increased by 16.8%, 24.6%, 8.3%, and 18.1%, respectively. These findings facilitate real-time tomato ripeness detection in complex greenhouse environments and provide perceptual information for subsequent management tasks such as harvesting.

## 1 Introduction

Tomato is the world’s second-largest vegetable in terms of cultivated area and the largest vegetable for processing (Quinet et al., 2019). It not only generates enormous economic value but also plays an irreplaceable role in the global food security system. Greenhouse tomatoes are a vital component of the international tomato industry and occupy a central position in the fresh-market sector (Ali et al., 2021). Notably, the fresh-market sector has an urgent need for timely harvesting: unripe tomatoes suffer from poor quality, which harms brand reputation, while overripe tomatoes are highly prone to decay. However, tomato harvesting currently relies heavily on manual labor. Facing labor shortages and an aging workforce (Wang et al., 2023; Kodsi et al., 2025), researchers and industry practitioners are attempting to gradually replace manual labor with robotic automated harvesting technology (Yao and Liu, 2024). This technology uses machine vision to rapidly and accurately identify tomato ripeness and locate fruits under complex environmental interference (Tang et al., 2020), guiding the end-effector to approach the fruit and perform grasping and harvesting actions, thereby achieving a fully automated harvesting process. Currently, the difficulty in determining the optimal ripeness window for tomato harvesting, combined with the complexity of recognition models that hinder practical deployment, prevents robotic harvesting from meeting the demands of efficient production.

Researchers have conducted extensive work in fruit and vegetable ripeness detection. Primary detection methods include spectral technology (Zhao et al., 2018), machine vision (Altaheri et al., 2019), dielectric properties, acoustic detection (Nicolaï et al., 2007), and electronic nose technology (Xu et al., 2015). Among these, machine vision is currently the most promising technological approach for real-time fruit ripeness detection. In the early stages, researchers applied traditional image recognition techniques to identify ripe fruits. For example, Liang et al. combined the minimum entropy criterion, median filtering, and the Otsu segmentation method to segment color tomato images under varying illumination, effectively eliminating lighting effects and accurately segmenting objects with distinct features (Liang and Jiang, 2014). However, that approach could only distinguish extreme ripeness levels and lacked robustness against interference. Subsequently, researchers further used features such as color, texture, and shape for identification. For instance, Zhuang et al. proposed a method to assess banana ripeness stages by evaluating different external optical characteristics (Zhuang et al., 2019). Using color features, the overall recognition accuracies of Naïve Bayes (NB), Linear Discriminant Analysis (LDA), and Support Vector Machine (SVM) classifiers were 99.2%, 100%, and 99.2%, respectively. Tu et al. applied an LLC-encoded RGB-DSIFT feature algorithm with a linear SVM classifier to recognize passion fruit RGB-D images of different ripeness levels in outdoor settings, achieving a detection accuracy of 92.71% and a ripeness classification accuracy of 91.52% (Tu et al., 2018).

With the development of deep learning, deep learning-based object detection algorithms represented by YOLO have been increasingly deployed in various agricultural robot vision tasks due to their superior performance. For instance, Ju et al. successfully applied an improved YOLOv5 model to an adaptive cruise weeding robot in paddy fields (Ju et al., 2024), achieving efficient identification of weeds. Meanwhile, deep-learning-based fruit ripeness detection methods have gradually been applied and promoted in recent years, as they can extract high-dimensional image features and are less susceptible to environmental interference. Mu et al. constructed a model based on a regional convolutional neural network and ResNet-101 that can automatically detect intact green tomatoes using deep learning, achieving an average accuracy and counting accuracy of 87.83% (Mu et al., 2020). To meet harvesting needs, Kim et al. proposed a deep learning network, Deep-ToMaToS, capable of simultaneous three-level ripeness classification and 6D pose (3D translation + 3D rotation) estimation of target fruits (Kim et al., 2022). They designed a harvesting motion control algorithm based on the ADD_S metric for 6D pose estimation, achieving a pose estimation accuracy of 96% and an average harvesting success rate of 84.5%. Zhao et al. proposed the OGS-YOLOv8 model for coffee bean ripeness recognition, achieving a detection accuracy of 73.7% and a good balance between high detection accuracy and low computational cost (Zhao and Wen, 2025). Although certain achievements have been made in ripeness detection research, the following scientific issues remain to be addressed when applied to complex greenhouse environments: (1) Existing methods have poor adaptability to complex lighting conditions in greenhouses, with limited ability to recover details in both bright and dark areas, making it difficult to ensure the stability of detection models across different lighting scenarios. (2) Under background interference from leaves, branches, etc., the edge details and effective features of fruit targets are easily overwhelmed by noise. The synergistic effects of existing attention mechanisms and residual structures in noise suppression and feature enhancement require further exploration. (3) In occlusion scenarios, there are long-range dependencies between fruit target regions. Expanding the receptive field helps improve the recognition rate of occluded targets, but directly increasing the convolution kernel size leads to a surge in computational load, contradicting the requirement for model lightweighting.

To address these issues, this study proposes an improved network model, DDC-YOLOv11n, based on YOLOv11n to achieve tomato ripeness detection under complex greenhouse conditions. The main contributions are as follows: (1) A highly robust tomato dataset covering various complex greenhouse scenarios was established, providing a data foundation for model training and evaluation. (2) The DDC-YOLOv11n network was designed: based on YOLOv11n, a Zero-DCE module was embedded at the input to achieve adaptive illumination enhancement; an improved DRSN was introduced in the shallow layers to suppress background noise; and an LKCBAM module was embedded in the deep layers to expand the receptive field, thereby optimizing feature extraction and fusion capabilities. (3) The detection performance of the improved model was verified through visualization analysis, ablation experiments, and comparative experiments.

## 2 Materials and methods

### 2.1 Image acquisition and dataset construction

**Figure 1:**
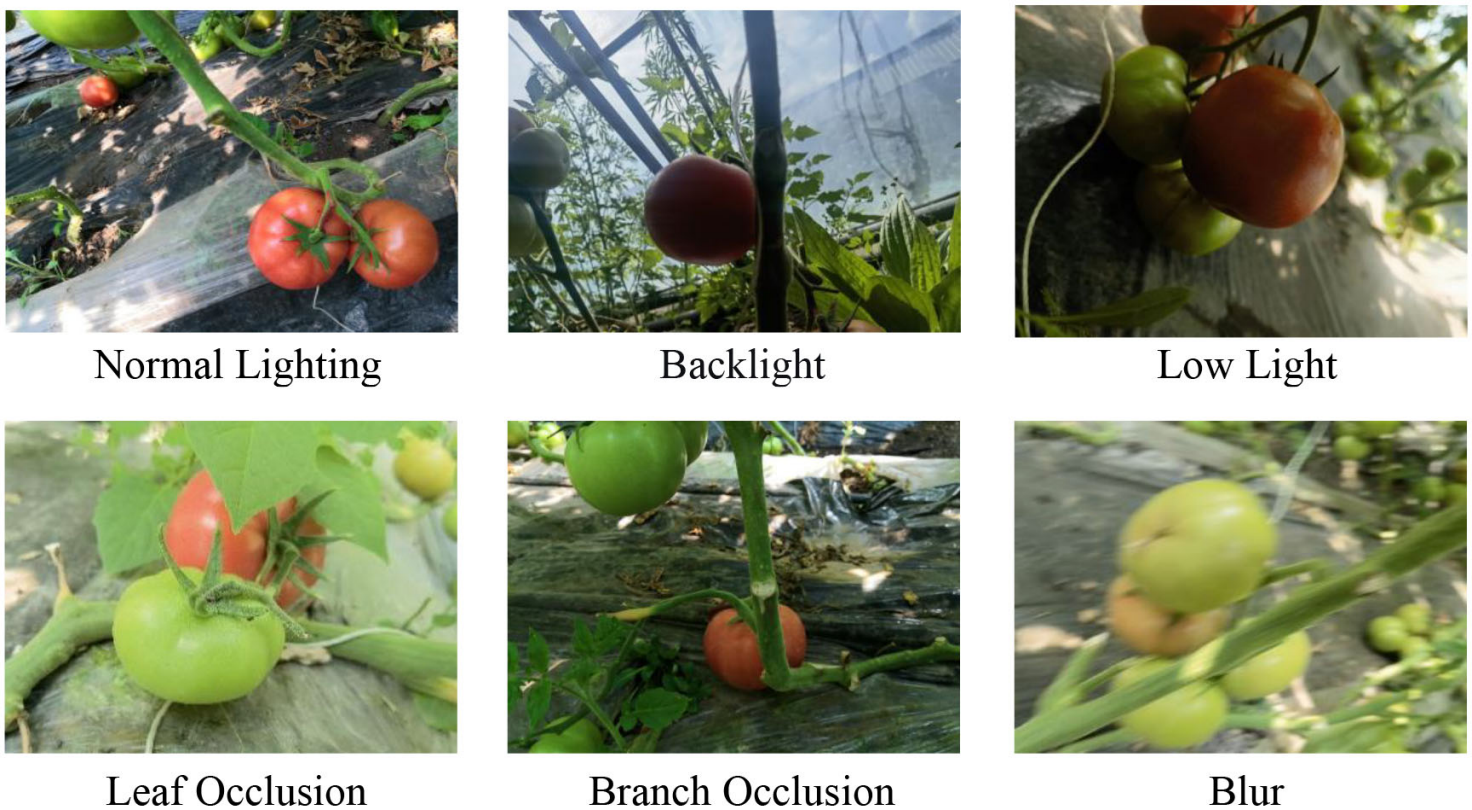
Tomato images in different scenarios.

We collected the RGB images used in this study from greenhouse tomatoes in Manas County, Changji Hui Autonomous Prefecture, Xinjiang Uygur Autonomous Region. Images were captured using a HUAWEI Mate40 Pro (NOH-AN00) device at two resolutions: 3072×4096 pixels and 3840×5120 pixels, and saved in JPG format. During shooting, the camera lens was oriented at random angles relative to the vertical plane, and the shooting distance was controlled within 20–50 cm. To improve the robustness of the image dataset, tomato fruit images were captured under various conditions, including normal lighting, branch and leaf occlusion, and fruit overlap (**Figure 1**), resulting in a total of 1739 images.

**Figure 2:**
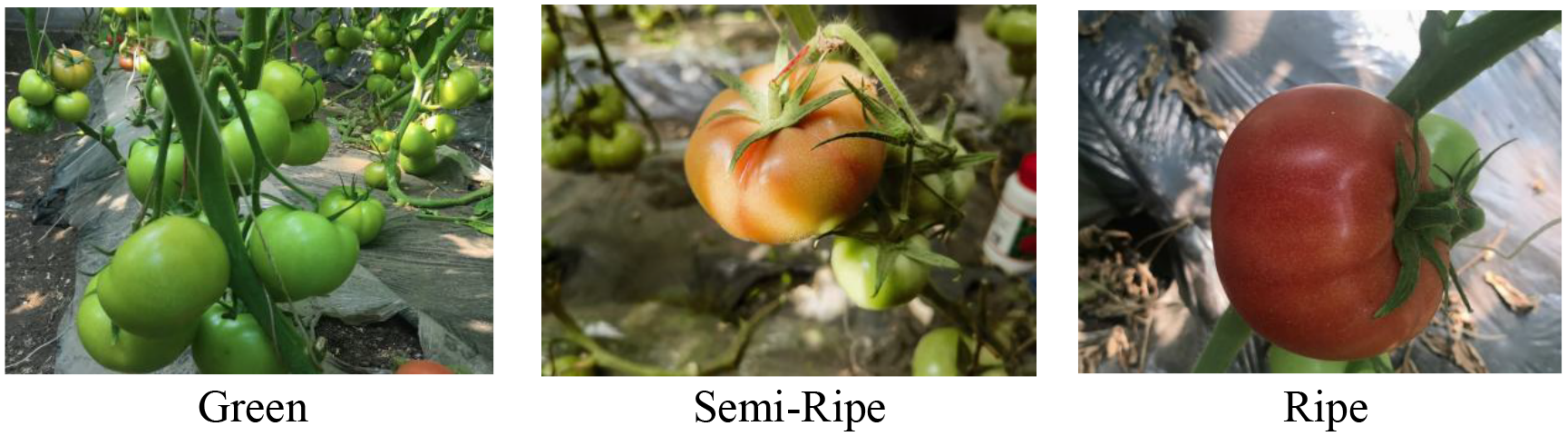
Tomatoes at different stages of ripeness.

**Figure 3:**
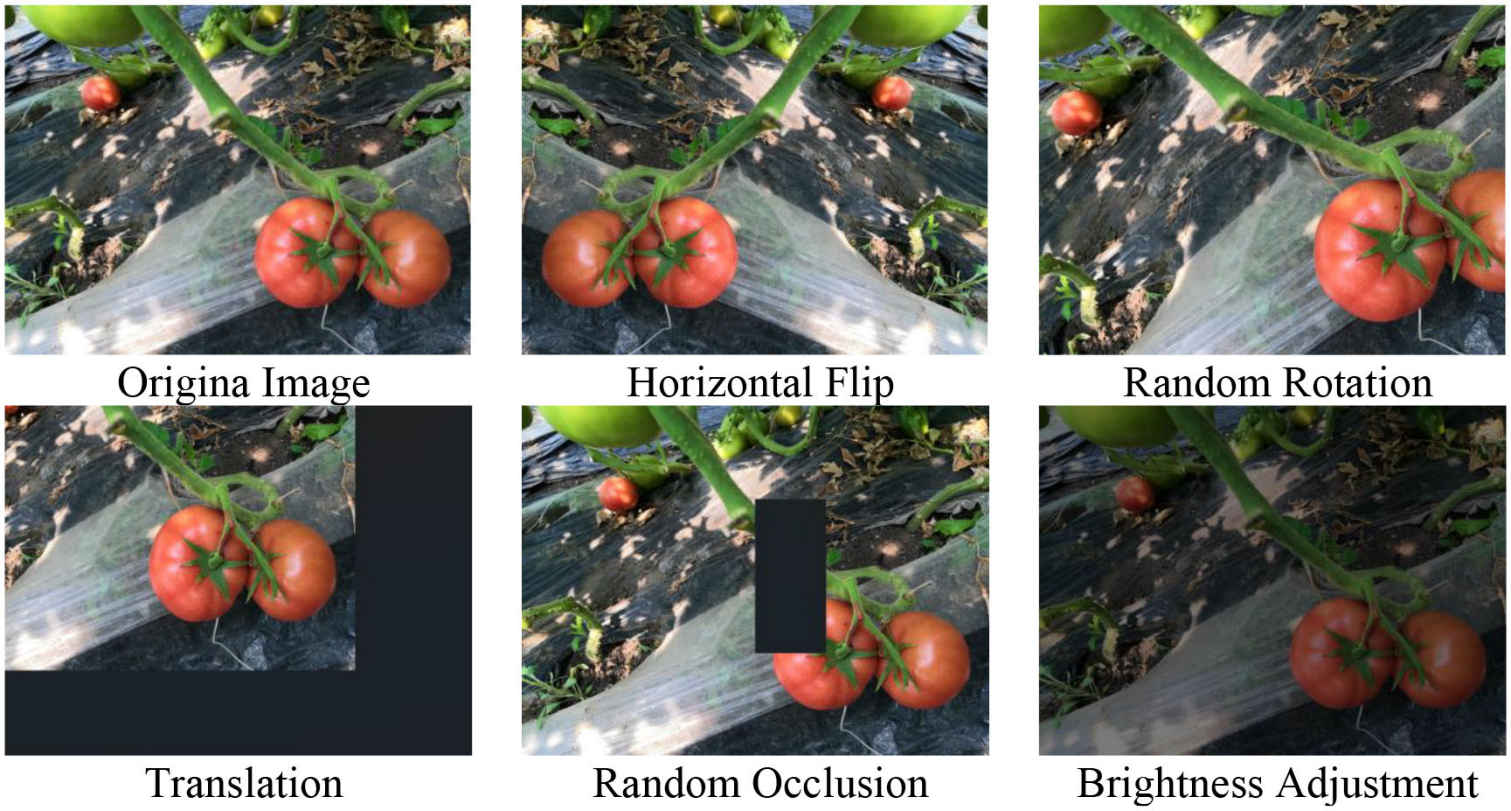
Data-augmented tomato images.

The open-source script X-AnyLabeling on GitHub was used to annotate the image dataset. Based on the actual growth conditions of tomatoes and production requirements (Radzevičius et al., 2009), three maturity classes were defined: fully ripe, semi-ripe, and green. The ripeness classification criteria were: fully ripe fruits have a red surface area ≥90%; semi-ripe fruits have a red surface area between 30% and 90%; green fruits have a red surface area between 0% and 30% (**Figure 2**). After running the X-AnyLabeling script, each tomato fruit image was labeled as one of three categories: ripe, semi_ripe, or green. For each image, an “xml” file containing the tomato fruit category and coordinate information was generated for training the neural network. In the “xml” file, xmin and ymin represent the coordinates of the top-left corner of the bounding box, and xmax and ymax represent the coordinates of the bottom-right corner. Additionally, data augmentation techniques (horizontal flipping, rotation, and color adjustment) were applied (**Figure 3**). After data augmentation, we split the dataset of 10,434 images into training (8,348 images), validation (1,043 images), and test (1,043 images) sets in an 8:1:1 ratio.

### 2.2 Construction of the DDC-YOLOv11n network model

(1) DDC-YOLOv11n Network Architecture

The Ultralytics team released YOLOv11 on September 30, 2024. As reviewed by Sapkota et al., YOLOv11 employs an improved backbone and neck architecture, thereby enhancing feature extraction capabilities to achieve more accurate object detection and better performance on complex tasks, while maintaining an optimal balance between accuracy and performance. It can be seamlessly deployed in various environments, including edge devices, cloud platforms, and NVIDIA GPU-supported systems (Sapkota et al., 2025). YOLOv11 has five distinct network models: YOLOv11n, YOLOv11s, YOLOv11m, YOLOv11l, and YOLOv11x, which differ in network depth and feature map width. Among these, YOLOv11n offers the fastest processing speed and the smallest model size. Considering the practical requirements for speed and lightweightness, this study adopted the YOLOv11n model.

**Figure 4:**
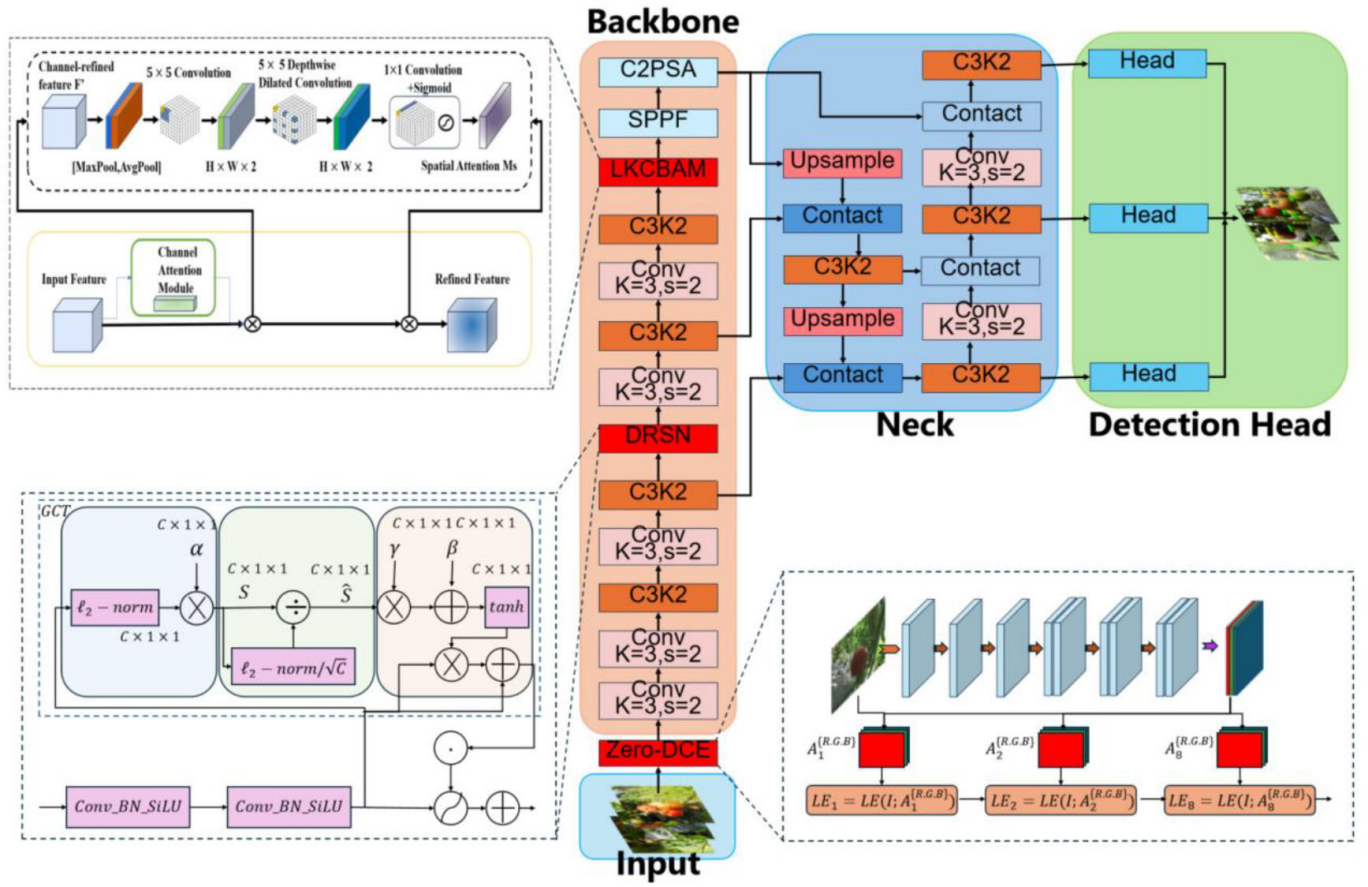
Network architecture of DDC-YOLOv11n.

We propose an improved YOLOv11n network architecture, DDC-YOLOv11n, which incorporates a Zero-DCE adaptive enhancement module, an improved DRSN, and an LKCBAM attention module. In summary, the Zero-DCE module, embedded at the input, adaptively corrects illumination distortions without the need for paired training samples training, thereby enhancing the brightness and detail fidelity of fruit images under low-light or strong-light conditions, laying the foundation for subsequent feature extraction. The improved DRSN and LKCBAM attention modules are introduced into the feature extraction network. The improved DRSN suppresses background noise interference through its residual shrinkage mechanism, enhances the extraction of effective features related to fruit ripeness, and improves the model’s robustness against complex backgrounds. The LKCBAM attention module is inserted into the feature fusion stage to optimize channel and spatial attention allocation, focusing on key feature regions of the fruit and mitigating misclassifications caused by the similarity of features across fruits of different ripeness levels. The improved network architecture is shown in **Figure 4**.

(2) Zero-DCE Adaptive Enhancement Module

Lighting conditions in greenhouses are influenced by weather, film refraction, and supplemental lighting, often resulting in overexposure or underexposure, which leads to the loss of shadow details and color cast issues. Such uneven images make it difficult for the detection module to correctly identify and classify targets, resulting in missed detections and misclassifications (Afifi and Brown, 2019). Zero-DCE (Zero-Reference Deep Curve Estimation) is a deep learning-based unsupervised low-light image enhancement method that can effectively adjust the brightness of overexposed photos, restore details in highlight regions, and improve overall clarity (Guo et al., 2020). It achieves coordinated processing of overexposed and underexposed regions by designing a bidirectionally adjustable LE curve. The basic quadratic curve for a single iteration is given in Equation 1:

(1) LE(I(x);α)=I(x)+αI(x)(1−I(x))

Where *LE*(·) is the light enhancement function, *I*(*x*) is the normalized pixel value, *α* is the brightness adjustment coefficient (*α* > 0: increase brightness; *α* = 0: decrease brightness; *α* = 0: no adjustment), and 1 − *I*(*x*) is the adaptive weighting factor.

The higher-order iteration curve is expressed in Equation 2:

(2) $$LE_{n}\left(x\right)=LE_{n-1}\left(x\right)+A_{n}\left(x\right)LE_{n-1}\left(x\right)\left(1-LE_{n-1}\left(x\right)\right)$$

Where *LE_n_*(*x*) is the output of the n-th iteration, *LE_n_*_−1_(*x*) is the output of the (n−1)-th iteration, *A_n_*(*x*) is the adjustment coefficient map for the n-th iteration, and *x* is the pixel position.

**Figure 5:**
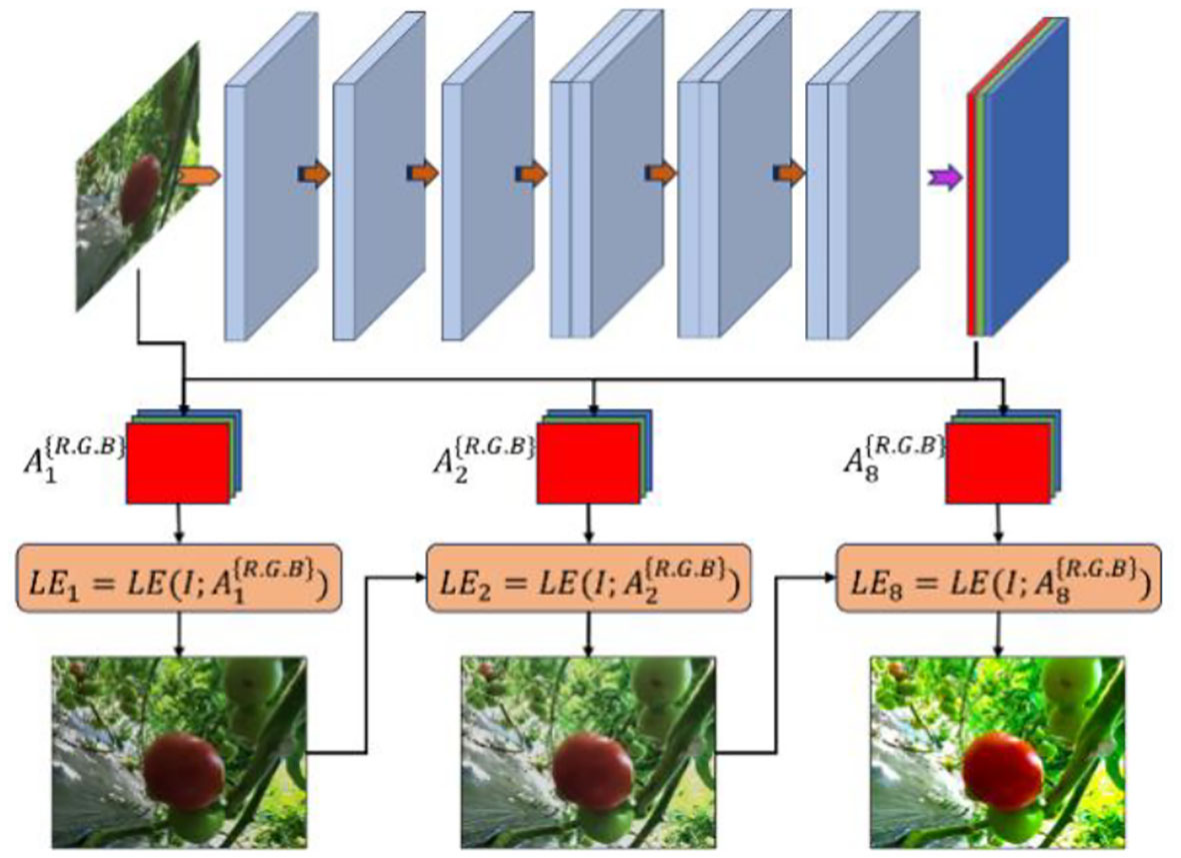
DCE-Net architecture and image iteration flowchart.

The parameter *A_n_*(*x*) is learned via a lightweight convolutional neural network, DCE-Net (Liu et al., 2025). DCE-Net consists of six identical 3×3 convolutional layers, and the feature map dimensions remain unchanged after convolution. The feature map from the last layer undergoes a 3×3 convolution followed by a Tanh activation, generating eight sets of final parameter maps. Each set of parameter maps is sequentially applied to the input RGB image. The resulting image after the iterations is median-filtered to serve as the final output, thereby suppressing the amplification of noise caused by enhancement. The DCE-Net architecture and image iteration workflow are shown in **Figure 5**.

DCE-Net achieves unsupervised training through four loss functions without the need for reference images. The total loss is calculated using Equation 3:

(3) $$L_{total}=L_{spa}+L_{exp}+W_{col}L_{col}+W_{tvA}L_{tvA}$$

Where *L_spa_* is the spatial consistency loss, *L_exp_* is the exposure control loss, *W_col_* and *L_col_* are the weight and loss for color constancy, and *W_tvA_* and *L_tvA_* are the weight and loss for illumination smoothness.

(3) Improved DRSN (Deep Residual Shrinkage Network)

The Deep Residual Shrinkage Network embeds an SE attention module branch within the convolutional layers to learn a set of thresholds, and then performs soft thresholding on the feature values along the main path. Soft thresholding is defined in Equation 4:

(4) $$y=\{\begin{array}{c} x-\tau \left(x>\tau \right)\\ 0\left(-\tau \leq x\leq \tau \right)\\ x+\tau \left(x<-\tau \right) \end{array}$$

Where *τ* is the set threshold. Noise suppression is achieved by setting feature values with absolute value less than *τ* to zero, while preserving the useful signal components.

**Figure 6:**
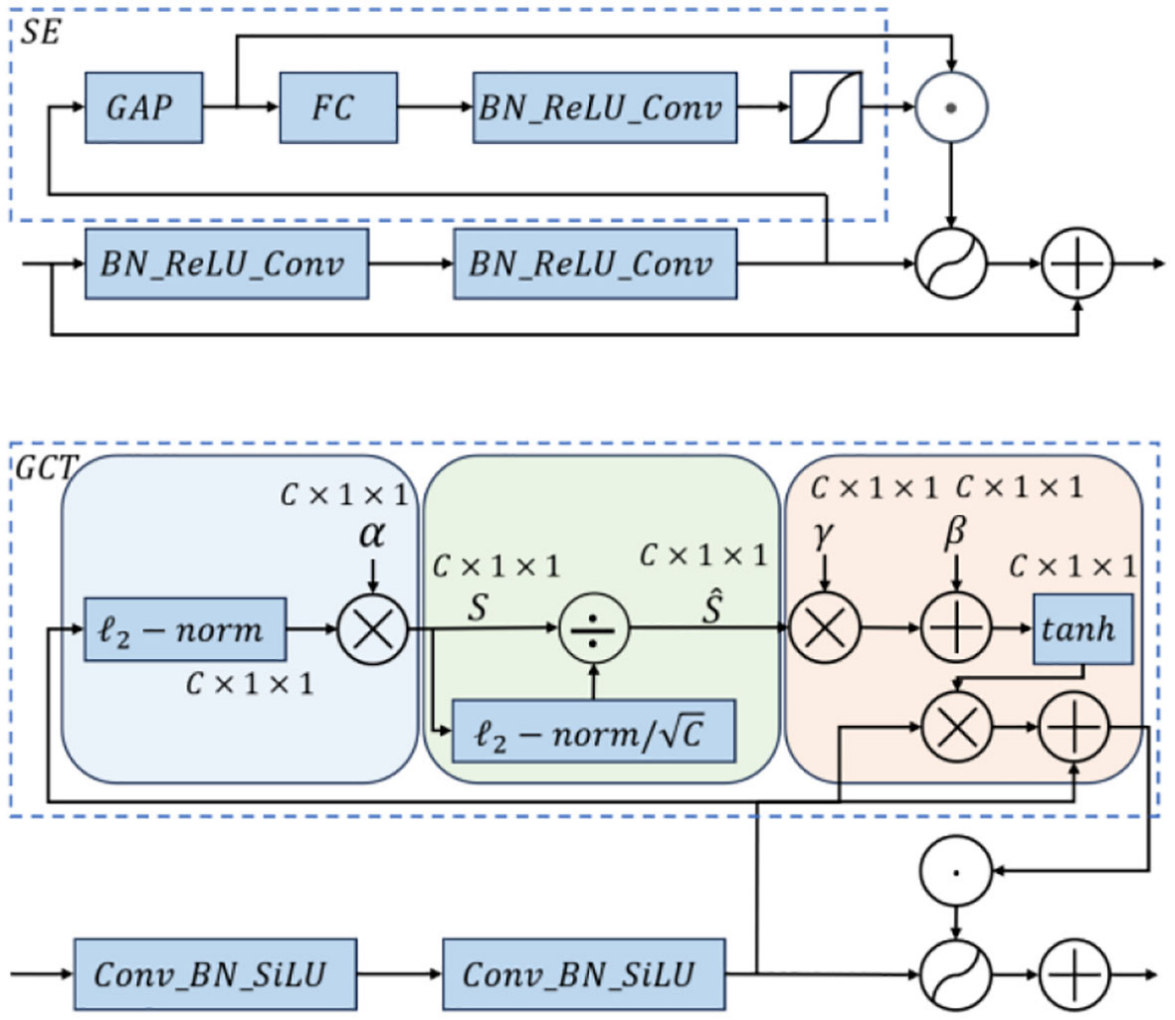
Structure of the improved DRSN module.

This study improves the network architecture of DRSN by replacing the SE module with a GCT module (Yang et al., 2020). The GCT module has fewer parameters and balances competition and cooperation through a gating mechanism, significantly enhancing noise robustness and task performance. The architecture is shown in **Figure 6**. Input features are first processed by two convolutional units, then fed into a channel attention mechanism. This mechanism adaptively learns and generates a set of weight parameters for calibrating channel responses through operations such as L2 normalization. Finally, these modulated weights are fused with the features from the backbone path, thereby dynamically enhancing the model’s ability to focus on key channel information and improving the efficiency and performance of the overall feature representation.

(4) Improved CBAM (Convolutional Block Attention Module)

The CBAM (Woo et al., 2018) is a simple yet efficient attention module for feedforward convolutional neural networks, achieving adaptive feature enhancement through dual-path weight learning. First, a channel attention map is generated using global average pooling and a fully connected layer to learn channel-level importance weights, focusing on key feature channels. Then, a spatial attention map is generated by channel compression and convolution to learn spatial-level weights, highlighting important regions in the feature map. The generated attention maps are sequentially applied to the input feature map. This design not only decouples channel and spatial attention but also ensures computational efficiency; with an extremely low number of parameters, it is particularly suitable for lightweight object detection networks.

Large-scale convolution kernels have a wide receptive field and strong global correlations, enabling them to cover a larger spatial range and capture long-range dependencies between objects. Small-scale convolution kernels focus on local details, have low computational complexity, and a small receptive field, allowing them to accurately capture fine-grained features such as edges and textures. The 7×7 convolution in the CBAM spatial attention module can only capture local spatial correlations, making it difficult to model long-range dependencies between tomato regions in complex orchard environments. However, further increasing the kernel size would lead to a significant increase in computational cost, conflicting with the lightweight requirements of real-time detection. This study replaces the original 7×7 standard convolution with a decomposed large-kernel convolution. Specifically, a 15×15 large-kernel convolution is decoupled into a combination of three cascaded components: a depthwise convolution, a depthwise dilated convolution with a dilation rate of 3, and a 1×1 pointwise convolution. Based on this structure, the Softmax activation function is reintroduced to convert the values mapped to each spatial location into saliency weights corresponding to spatial features, thereby achieving adaptive alignment with the channel attention output (Hou et al., 2022). The decomposition process is as follows.

Assuming the number of kernels in the large-kernel convolution is *K*, The number of depthwise convolution kernels is given by Equation 5:

(5) $$K_{DW-Conv}=\frac{K}{d}$$

The number of depthwise dilated convolution kernels is given by Equation 6:

(6) $$K_{DW-Conv}=\frac{K}{d}$$

Where *d* is the dilation rate of the depthwise dilated convolution.

The output of the improved spatial attention module is expressed in Equation 7:

(7) $$Y_{s}\left(X\right)=\text{σ}\left(f_{Conv}^{1\times 1}\left(f_{Conv-D-Conv}^{5\times 5}\left(f_{DW-Conv}^{5\times 5}\cdot \left(\left[\text{Avgpool}vgpool\left(X\right);\text{Maxpool}\left(X\right)\right]\right)\right)\right)\right)$$

Where $f_{Conv}^{1\times 1}$ is a 1×1 pointwise convolution, $f_{Conv-D-Conv}^{5\times 5}$ is a 5×5 depthwise dilated convolution with dilation rate 3, and $f_{DW-Conv}^{5\times 5}$ is a 5×5 depthwise convolution.

**Figure 7:**
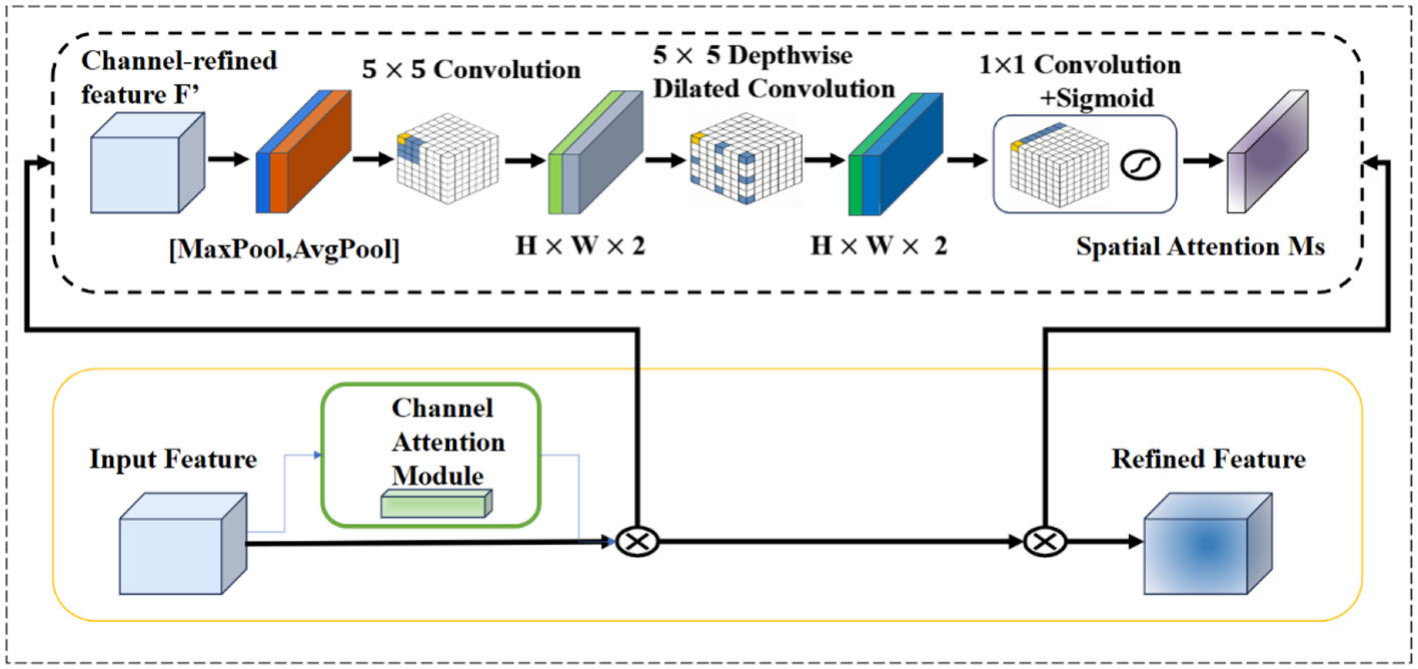
Schematic diagram of the LKCBAM network architecture.

By integrating the improved spatial attention module into CBAM, we obtain the Large Kernel Convolutional Block Attention Module (LKCBAM) (Ren et al., 2025), whose structure is shown in **Figure 7**.

Specifically, this module employs a three-branch parallel architecture. First, a 5×5 depthwise convolution accurately captures local details, with a computational cost that is only one-tenth that of standard convolution. Concurrently, a 5×5 depthwise dilated convolution with a dilation rate of 3 is introduced; through dilated sampling, the effective receptive field is expanded to 13×13, enabling the model to span fruit regions obscured by branches and leaves. Finally, a 1×1 pointwise convolution lightly fuses the features from the two branches and adjusts the dimensionality. The collaborative output of these three components is passed through a Sigmoid activation to generate a spatial weight map, ultimately achieving an equivalent receptive field of 17×17 with only a small increase in computational cost.

### 2.3 Software and hardware configuration

We ran all detection models under identical hardware and software configurations to minimize the adverse effects of runtime environments and other factors on the results, ensuring accuracy and reliability. The hardware configuration was as follows: Intel^®^ Core™ i9-14900K at 6.0 GHz, 62 GB DDR5 RAM, and an NVIDIA GeForce RTX 5090 discrete graphics card. CUDA 12.8, CUDNN 9.1, Python 3.11.14, and PyTorch 2.9.1+cu128 were used to build the deep learning environment. The model training parameters were: initial learning rate 0.01, weight decay coefficient 0.0005, and 200 epochs. A batch size of 64 was used to improve detection performance. All models were trained and evaluated with an input image size of 640×640 pixels. Automatic Mixed Precision (AMP) was enabled during training to reduce memory usage and accelerate computation.

### 2.4 Ablation and comparative experiments

This study designed an approach that first uses Zero-DCE for preprocessing and then integrates DRSN and LKCBAM into the backbone. To fully demonstrate the superiority of these improved models and evaluate their interactions, we conducted ablation experiments to compare and analyze the performance of the models before and after the improvements. The models used in the ablation experiments and the corresponding added modules are shown in **Table 1**. In addition to ablation experiments on YOLOv11n with added modules, we designed comparative experiments between DDC-YOLOv11n and three classic network architectures (YOLOv5n, YOLOv8n, and YOLOv10n) to further verify whether DDC-YOLOv11n achieves good tomato ripeness recognition and classification performance under the research conditions described in this paper.

**Table 1:** Models for ablation experiments.

Experimental group	YOLOv11n	Improved module		
		Zero-DCE	DRSN	LKCBAM
YOLOv11n	✓	–	–	–
Group A	✓	✓	–	–
Group B	✓	–	✓	–
Group C	✓	–	–	✓
Group D	✓	✓	✓	–
Group E	✓	✓	–	✓
Group F	✓	–	✓	✓
DDC-YOLOv11n	✓	✓	✓	✓

### 2.5 Evaluation metrics

In this study, we employed several commonly used performance evaluation metrics in deep learning to validate the model and assess the feasibility of improving YOLOv11 for tomato ripeness detection. Relevant metrics include Precision, Recall, F1-Score, mean average precision (mAP), number of parameters, and floating-point operations (FLOPs). The definitions of Precision, Recall, and F1-Score are based on the work of Padilla et al. (2020).

Precision is defined in Equation 8:

(8) $$Precision=\frac{TP}{TP+FP}$$

Where TP is the number of true positives and FP is the number of false positives.

Recall is defined in Equation 9:

(9) $$Recall=\frac{TP}{TP+FN}$$

Where FN is the number of false negatives.

The F1-Score is the harmonic mean of precision and recall, as shown in Equation 10:

(10) $$F1-Score=2\times \frac{Precision\times Recall}{Precision+Recall}$$

The F1-Score is the harmonic mean of precision and recall, providing a comprehensive assessment of a model’s ability to accurately identify objects and its rate of missed detections. A higher F1-Score indicates a better balance between these two metrics. Meanwhile, the mean average precision (mAP) is the average of the average precision (AP) values across all categories, characterizing the model’s overall performance in multi-class detection tasks.

The number of parameters (Parameters) is the total count of all trainable weights in the model, which directly determines storage requirements and the potential risk of overfitting; it is one of the metrics for assessing model lightweightness.

FLOPs (floating-point operations) represent the number of floating-point operations required for a single forward inference, a key indicator of computational complexity. Lower FLOPs indicate higher operational efficiency on resource-constrained devices.

## 3 Results and analysis

### 3.1 Ablation experiment results

#### 3.1.1 Analysis of loss function convergence

**Figure 8:**
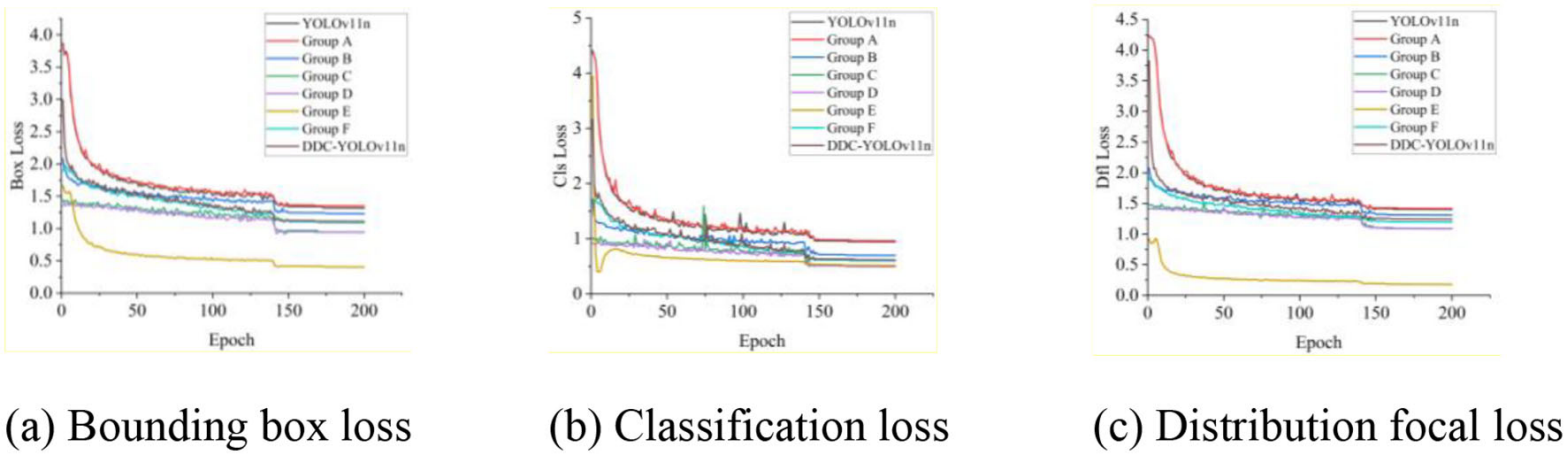
Training loss curves of different improved network models: **(a)** bounding box loss, **(b)** classification loss, **(c)** distribution focal loss.

Both the original YOLOv11n and the seven improved models were trained for 200 epochs; the loss function convergence during training is shown in **Figure 8**. Horizontally, Groups C and  D exhibited relatively flat trends for all three loss functions at the early training stage compared to the other six groups, with a smaller decrease throughout training; the remaining six groups showed a rapid decline in all three loss functions during the first 30 epochs, indicating fast model convergence. Overall, for all eight groups, the three loss functions remained relatively flat between epochs 30 and 140, with a slight decline with a slight decline occurring in the final stage between epochs 140 and 200. Vertically, YOLOv11n, Group A, DDC-YOLOv11n, and Group E (during classification loss) showed the largest decreases. The largest drop was observed in the distribution-focal loss of YOLOv11n and Group A, which decreased by 3.4. It can be observed that during the training of Group E, there was a noticeable rapid drop followed by a rapid rise in the first 0–10 epochs, suggesting that the two modules in Group E had not yet begun to operate synergistically. During the classification loss decline, YOLOv11n, Group C, and DDC-YOLOv11n exhibited several minor fluctuations between epochs 60 and 100, indicating the presence of data samples that were slightly more challenging for these three models. By observing the overall loss decline, it can be seen that none of the eight models exhibited underfitting or overfitting.

#### 3.1.2 Analysis of ablation experiment results

**Figure 9:**
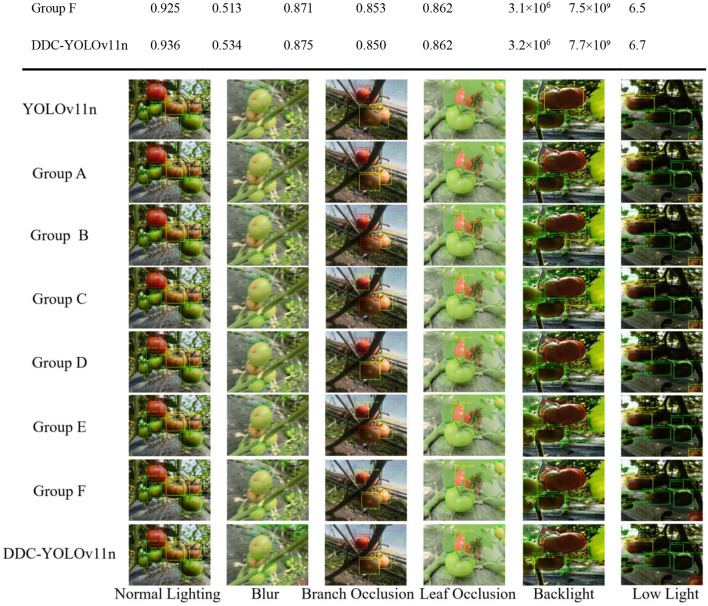
Performance of different improved network models in recognizing different ripeness levels across multiple scenarios.

The results of the ablation experiments are shown in **Table 2** and **Figure 9**. After Zero-DCE low-light preprocessing, Group A achieved an improvement of about 12.5% in recall and mAP@0.5, and about 12.4% in mAP@0.5:0.95 and precision, compared to YOLOv11n. This demonstrates that Zero-DCE can adaptively enhance image brightness and contrast without relying on prior data, effectively improving target visibility under low-light and shadow-occluded conditions, thereby enhancing the network’s ability to extract target edges and texture features, which is highly beneficial for multi-scenario target recognition and tomato ripeness classification. Under varying lighting, the model incorporating Zero-DCE was able to more accurately identify tomatoes of different ripeness levels than the other two single-module models. After introducing the DRSN module, Group B improved precision by 22.8%, recall by 8.9%, and mAP@0.5 by 14.6% compared to YOLOv11n, indicating that Group B’s detail-enhancing feature extraction is more suitable for our research scenario than Group A’s illumination preprocessing only. Compared to the original YOLOv11n and the model with LKCBAM alone, DRSN detected more tomatoes in low-light conditions and when combined with Zero-DCE. After introducing the LKCBAM module, all metrics of Group C improved to varying degrees: mAP@0.5 increased by 13.4%, precision by 17.1%, and recall by 7.4%. This is because the combination of depthwise and depthwise dilated convolutions helps extract detailed features of tomatoes and establishes long-range dependencies between tomatoes by expanding the receptive field, thereby enhancing both precision and recall. Compared to the other groups, Group C detected tomatoes at greater distances. When two modules were combined, the performance was better than using a single module. Among the two-module combinations, the network with DRSN and LKCBAM performed best, achieving increases in precision, recall, and mAP@0.5 of 24.1%, 8.7%, and 15.5%, respectively, relative to YOLOv11n. This may be attributed to the simultaneous enhancement of both fine-grained and wide-view feature extraction. The model with all three modules integrated achieved the best performance across all tomato ripeness classification scenarios, detecting more tomatoes and classifying their ripeness more accurately than the other groups. It is worth noting that the mAP@0.5:0.95 of DDC-YOLOv11n (0.534) is slightly lower than that of Group B (0.540). One possible reason is that while LKCBAM expands the receptive field (equivalent to 17×17) to capture long-range dependencies between occluded fruits, it may introduce slight fluctuations in bounding box localization accuracy when aggregating information over a larger spatial range. In contrast, Group B relies only on DRSN denoising, so the spatial resolution of the feature maps remains unaffected, resulting in more accurate bounding box regression.

**Table 2:** Comparison of ablation experiment results.

Model configuration	mAP@0.5	mAP@ 0.50:0.95	Precision	Recall	F1-Score	Params (M)	FLOPS (G)	Model size (MB)
YOLOv11n	0.801	0.421	0.702	0.785	0.73	2.6×10^6^	6.5×10^9^	5.5
Group A	0.901	0.473	0.789	0.883	0.833	2.7×10^6^	6.7×10^9^	5.7
Group B	0.918	0.540	0.862	0.855	0.860	2.9×10^6^	7.1×10^9^	6.1
Group C	0.908	0.479	0.822	0.843	0.834	2.8×10^6^	6.9×10^9^	5.9
Group D	0.920	0.521	0.864	0.861	0.862	3.0×10^6^	7.3×10^9^	6.3
Group E	0.921	0.511	0.851	0.872	0.861	2.9×10^6^	7.1×10^9^	6.1
Group F	0.925	0.513	0.871	0.853	0.862	3.1×10^6^	7.5×10^9^	6.5
DDC-YOLOv11n	0.936	0.534	0.875	0.850	0.862	3.2×10^6^	7.7×10^9^	6.7

#### 3.1.3 Heatmap results of attention mechanisms

**Figure 10:**
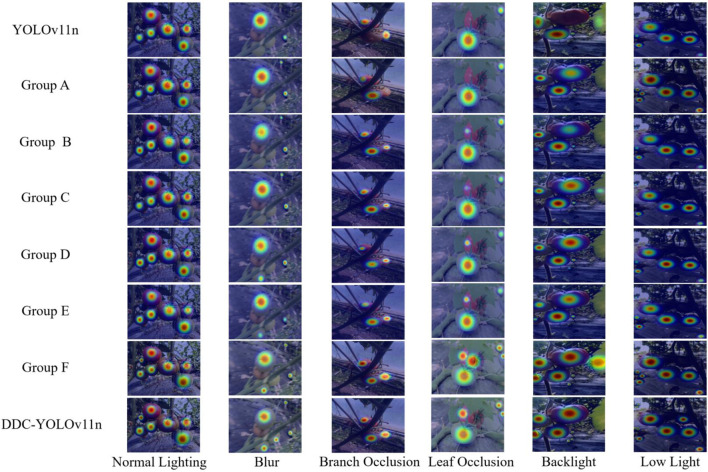
Comparison of Grad-CAM visualization results for different improved models across various scenarios.

Grad-CAM is a visualization method that intuitively displays the importance weights of various image features in the input image through gradient averaging, without modifying the network architecture or retraining the model. The heatmap visualization results for each improved model are shown in **Figure 10**. Whether the three modules (Zero-DCE, LKCBAM, DRSN) were integrated individually, in pairs, or all together, they all outperformed the original YOLOv11n when dealing with blurry images, successfully detecting more tomato targets. Comparing the heatmaps when different modules were integrated into the P2 and P4 layers, the DRSN attention module at the P2 layer could remove redundant noise, enabling the small-object detection layer to better identify tomatoes. The LKCBAM module at the P4 layer established long-range dependencies between two distant target regions, making the connections between tomato pixel regions tighter and the distinction between tomato and background regions more pronounced. When pairing Zero-DCE with DRSN or LKCBAM, both combinations achieved good results in low-light scenes. When all three modules were integrated, the performance was slightly better than the other model groups.

### 3.2 Analysis of comparative experiment results

To fully validate the superiority of the improved network, we compared YOLOv5n, YOLOv8n, YOLOv10n, and YOLOv11n with DDC-YOLOv11n. As shown in **Table 3**, although DDC-YOLOv11n has increased parameters and computational complexity compared to the traditional models, its precision improved by 17.6%, 19.7%, and 28.3% over YOLOv5n, YOLOv8n, and YOLOv10n, respectively; recall improved by 14.2%, 16.4%, and 18.7%, respectively, significantly alleviating missed detections. The F1-Score reached 86.2%, demonstrating a better balance between precision and recall. The improvements in mAP@0.5 were also significant, increasing by 19.1%, 17.7%, and 25.5%, respectively, indicating stronger generalization ability.

**Table 3:** Comparison of experimental results between the improved YOLOv11n model and classic models.

Model	mAP@0.5	mAP@ 0.5:0.95	Precision	Recall	F1-Score	Params (M)	FLOPs (G)	Model size (MB)
YOLOv5n	0.786	0.379	0.744	0.744	0.71	2.5×10^6^	7.1×10^9^	5.3
YOLOv8n	0.795	0.355	0.731	0.73	0.70	3.0×10^6^	8.1×10^9^	6.3
YOLOv10n	0.746	0.374	0.682	0.716	0.66	2.3×10^6^	6.5×10^9^	5.8
DDC-YOLOv11n	0.936	0.534	0.875	0.850	0.862	3.2×10^6^	7.7×10^9^	6.7

**Figure 11:**
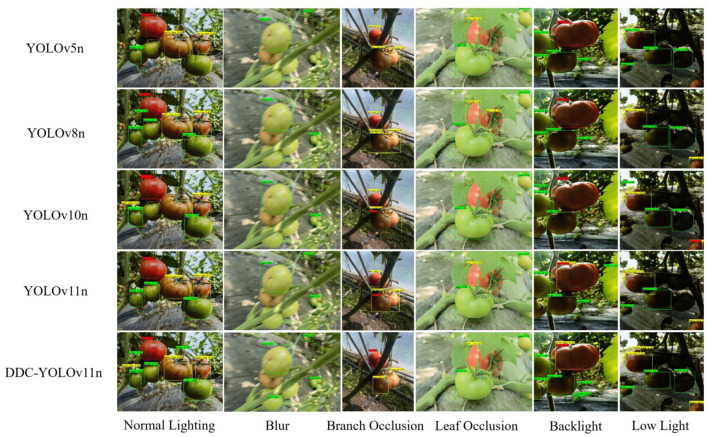
Comparison of recognition performance between the improved YOLOv11n and classic models under different scenarios.

To verify the actual detection performance of the improved YOLOv11n network, we tested images from the test set using YOLOv5n, YOLOv8n, YOLOv10n, YOLOv11n, and the best-performing DDC-YOLOv11n model (**Figure 11**). In standard tomato ripeness detection, YOLOv5n, YOLOv8n, YOLOv10n, and YOLOv11n all exhibited varying degrees of missed detections. In contrast, DDC-YOLOv11n detected more tomatoes with high confidence. In tomato ripeness detection, YOLOv5n, YOLOv8n, YOLOv10n, and YOLOv11n also exhibited varying degrees of false positives. When obstructed by branches, they misclassified ripe tomatoes as semi-ripe; in low-light conditions, YOLOv5n detected semi-ripe tomatoes as green. The integration of the three modules reduces redundant features during detection, enabling the final improved model to detect tomatoes more effectively and determine their ripeness more accurately.

## 4 Discussion

This study addresses three core challenges in real-time tomato ripeness detection under complex greenhouse environments—image detail recovery under complex lighting, effective feature extraction under background noise interference, and modeling long-range dependencies in occlusion scenarios—by proposing an improved DDC-YOLOv11n model. By embedding the Zero-DCE module at the input of YOLOv11n and introducing the improved DRSN and LKCBAM modules into the backbone network, the model significantly improves detection accuracy while maintaining high computational efficiency. This section analyzes the working mechanisms of each improved module, examines the model’s performance across various challenging scenarios, compares it with existing research, and finally discusses limitations and future work.

### 4.1 Impact of each improved module on tomato ripeness detection performance

Ablation results show that the Group A model, which incorporates the Zero-DCE module, achieves a 12.5% improvement in mAP@0.5 over the original YOLOv11n (**Table 2**). This improvement is visually confirmed in the Grad-CAM heatmap (**Figure 10**): under low-light and backlit conditions, Group A exhibits a more focused attention region on the tomato fruit with clearer boundaries. These results validate the effectiveness of the Zero-DCE module. Its core advantage lies in its zero-reference learning strategy (Guo et al., 2020), which enables the lightweight DCE-Net network to estimate a series of pixel-level brightness adjustment curves for dynamic image correction without paired normal/low-light images. Compared to traditional methods based on histogram equalization or gamma correction, Zero-DCE recovers detail information in overexposed highlights and underexposed shadows in a data-driven manner, avoiding local information loss caused by global enhancement. In greenhouse environments, lighting varies with time (day/night), weather (cloudy/sunny), and human factors (supplemental lights, film shading). The introduction of this module provides more consistent image features at the input stage, laying a solid foundation for subsequent feature extraction and object recognition. This finding aligns with the conclusion of Liu et al. (2025) that the unsupervised Zero-DCE network effectively improves the visual quality of low-light images, thereby aiding downstream vision tasks.

In greenhouse scenarios, background elements such as leaves, branches, and soil intertwine with fruit targets, creating significant noise interference that severely affects feature purity. After introducing the improved DRSN module into Group B, precision increased by 22.8% and recall by 8.9% compared to YOLOv11n (**Table 2**), indicating that this module performs excellently in suppressing false positives and reducing false negatives. The original DRSN learns and filters out irrelevant information in feature maps by combining the SE attention mechanism with soft thresholding (Zhao et al., 2020). Replacing the SE module with the GCT module (Yang et al., 2020) was key to enhancing efficiency and performance. The GCT module establishes a balance between competition and cooperation across channels through a gating mechanism; it has fewer parameters but can more accurately identify and suppress noise features while enhancing key features related to tomato ripeness, such as texture, color, and shape. As shown in the heatmap (**Figure 10**), Group B effectively reduces responses in background areas when dealing with blurry or branch-occluded images, allowing attention to focus more on the fruit itself. This ability to finely separate “signal” from “noise” is the reason why deep residual shrinkage networks perform exceptionally well in tasks with low signal-to-noise ratios, such as fault diagnosis (Zhao et al., 2020); this study demonstrates their great potential in agricultural computer vision.

In scenarios where fruits overlap or are severely occluded by branches and leaves, long-range dependencies between objects are crucial for accurate identification. After incorporating LKCBAM, Group C achieved improvements in all metrics, with mAP@0.5 reaching 90.8% and precision 82.2% (**Table 2**). Compared to the 7×7 spatial convolution of the original CBAM, LKCBAM employs a decomposed large-kernel convolution strategy (Hou et al., 2022) to expand the equivalent receptive field to 17×17 while controlling the increase in computational complexity. This design draws on the idea of dilated convolution: first, a 5×5 depthwise convolution captures local details; then, a 5×5 depthwise dilated convolution with dilation rate 3 performs sparse sampling, equivalent to a 13×13 convolution kernel; finally, channel fusion is achieved via a 1×1 pointwise convolution. This decomposition enables the model to overcome physical occlusions from branches and leaves, establishing semantic associations between different tomato fruit regions. As shown in the heatmap (**Figure 10**), Group C generates continuous and highly highlighted response regions for two or more occluded tomato fruits, demonstrating its ability to capture long-range object correlations. This improvement aligns with the approach of Ren et al. in tomato detection (Ren et al., 2025), where large-kernel attention expands the receptive field, effectively mitigating the insufficient perception of traditional small convolution kernels in occlusion scenarios.

### 4.2 Overall performance and superiority of the DDC-YOLOv11n model

Ablation results show that the DDC-YOLOv11n model, which integrates Zero-DCE, improved DRSN, and LKCBAM, achieves optimal performance across all evaluation metrics. mAP@0.5 reaches 93.6%, and precision, recall, and F1-Score are 87.5%, 85.0%, and 86.2%, respectively (**Table 2**). Compared to the original YOLOv11n, mAP@0.5 improves by 16.8% and precision by 24.6%, fully demonstrating the synergistic enhancement effect of the three modules. The Zero-DCE module performs adaptive illumination correction, providing the subsequent network with illumination-robust feature representations; the improved DRSN module effectively suppresses background noise during early feature extraction, thereby purifying the feature space; and the LKCBAM module expands the receptive field during feature fusion to enhance the recognition of occluded targets. Together, they form a complete optimization chain from data preprocessing to feature purification to feature enhancement.

Comparative experiments with mainstream lightweight models such as YOLOv5n, YOLOv8n, and YOLOv10n further validate the superiority of DDC-YOLOv11n. Although its parameter count (3.2 M) and computational cost (7.7 GFLOPs) have increased, this is a reasonable trade-off for improving the model’s adaptability to complex scenarios. The significant improvements in precision, recall, and mAP (**Table 3**) demonstrate that the additional computational resources yield a comprehensive leap in detection performance, which is crucial for harvesting robots that must balance real-time operation with accuracy. The actual detection results (**Figure 11**) clearly show that DDC-YOLOv11n achieves higher detection confidence and lower false-positive/false-negative rates across various scenarios (normal, occluded, low-light), and performs particularly robustly in distinguishing ripeness levels. For example, in low-light conditions, YOLOv5n misclassifies semi-ripe tomatoes as green, while DDC-YOLOv11n correctly identifies them, thanks to the brightness correction of Zero-DCE and the noise suppression of DRSN.

### 4.3 Comparison with existing tomato ripeness detection studies

We compare our results with recent studies on tomato ripeness detection. Mu et al. (2020) used Faster R-CNN and ResNet-101 to detect highly occluded green tomatoes, achieving an average accuracy of 87.83%, but their model is too complex to meet real-time requirements. Kim et al. proposed Deep-ToMaToS, which can simultaneously perform ripeness classification and 6D pose estimation (Kim et al., 2022), but its network structure is complex and difficult to train. Liu et al. developed a YOLOv3-based tomato detection model that performs well in simple scenarios but lacks robustness in complex environments (Liu et al., 2020). Wang et al. improved Faster R-CNN by introducing an attention mechanism to enhance multi-object detection in complex scenarios (Wang et al., 2022), but detection speed remains a bottleneck. In contrast, the YOLOv11n baseline selected in this study already strikes a good balance between speed and accuracy (Sapkota et al., 2025), and DDC-YOLOv11n significantly improves detection accuracy in complex greenhouse environments through careful module design with virtually no sacrifice in speed.

Recent studies have also explored similar improvement approaches. For example, Hao et al. improved tomato ripeness detection by refining the BiAttFPN fusion architecture and YOLOv11-SLBA modeling (Hao et al., 2025); Yao et al. proposed an Edge-guided DETR model that uses edge information to guide Transformer detection (Yao et al., 2025); Wang et al. proposed SWMD-YOLO (Wang et al., 2025), and Wang et al. proposed a lightweight algorithm based on an improved RT-DETR (Wang et al., 2024), both making progress in greenhouse tomato detection. Compared with these works, our method features a more systematic multi-module collaboration: Zero-DCE tackles illumination at the source, the improved DRSN suppresses noise during shallow feature extraction, and LKCBAM expands the receptive field during deep feature fusion. Each module fulfills its specific role, collectively addressing the multiple challenges posed by greenhouse environments. This optimization strategy provides a new reference for solving object detection problems in complex agricultural scenarios.

### 4.4 Limitations and future prospects

Although DDC-YOLOv11n achieves excellent detection performance in complex greenhouse environments, certain limitations remain. First, the model’s computational complexity and parameter count have increased compared to baseline models (**Table 2**). While this increase is negligible for workstations equipped with high-performance GPUs such as the RTX 5090, it may still pose a real-time bottleneck for actual harvesting robots deployed on edge computing devices like the Jetson. Future work should explore lightweight techniques such as model pruning and knowledge distillation to further reduce model size while maintaining detection accuracy, meeting the stringent requirements of practical deployment.

Second, although the dataset covers a variety of complex scenarios, we collected it exclusively in greenhouses in a single region, and it features a relatively limited range of tomato varieties. The model’s generalization ability across different regions, cultivation methods (e.g., open-field, hydroponics), tomato varieties, and growing seasons remains to be verified. Future work should further expand the diversity and scale of the dataset and conduct cross-domain transfer experiments to comprehensively evaluate the model’s robustness.

Finally, the detection tasks in this study are limited to ripeness classification and fruit localization. In actual robotic harvesting operations, in addition to the fruit’s position and ripeness, more detailed information such as the fruit’s 3D spatial pose and the position of the stem is needed to guide the robotic arm for non-destructive harvesting. Therefore, future work could extend the DDC-YOLOv11n model to a multi-task network or deeply integrate it with an RGB-D camera to achieve an end-to-end solution from detection to grasping.

## 5 Conclusion

For tomato ripeness detection tasks across different scenarios, we introduced the Zero-DCE preprocessing module, an improved DRSN attention module, and an enhanced CBAM spatial attention module. By integrating these modules into the YOLOv11n network and comparing their recognition performance, we proposed the DDC-YOLOv11n network with an attention mechanism for tomato ripeness recognition in complex environments. Through this study, the following conclusions were drawn.

Ablation results indicate that, compared to the original YOLOv11n, DDC-YOLOv11n achieves a comprehensive improvement in detection performance while incurring only a slight increase in computational load. Specifically, mAP@0.5 increased by 16.8%, precision by 24.6%, and recall and F1-Score by 8.3% and 18.1%, respectively, validating the effectiveness of the proposed module combination.

The Grad-CAM heatmap visualization results qualitatively validate the mechanisms of each module: Zero-DCE focuses on illumination correction, DRSN suppresses background noise, and LKCBAM enhances cross-scale correlations for occluded targets.

Compared with mainstream lightweight detection models such as YOLOv5n, YOLOv8n, and YOLOv10n, DDC-YOLOv11n achieves the best detection accuracy (mAP@0.5: 93.6%) while maintaining low computational complexity, confirming its applicability in complex greenhouse scenarios.

Through the above experimental analysis, the proposed improved model demonstrates excellent performance in tomato ripeness detection across various greenhouse scenarios. Future research could further explore deployment optimization of the model on embedded platforms (such as the Jetson series) and its extension to more fruit and vegetable ripeness detection tasks, thereby advancing the practical development of smart agricultural equipment.

## Data Availability

The raw data supporting the conclusions of this article will be made available by the authors, without undue reservation.
